# Case report: Local bacteriophage therapy for fracture-related infection with polymicrobial multi-resistant bacteria: hydrogel application and postoperative phage analysis through metagenomic sequencing

**DOI:** 10.3389/fmed.2024.1428432

**Published:** 2024-07-12

**Authors:** Volker Alt, André Gessner, Maya Merabishvili, Florian Hitzenbichler, Gopala Krishna Mannala, David Peterhoff, Nike Walter, Jean-Paul Pirnay, Andreas Hiergeist, Markus Rupp

**Affiliations:** ^1^Department of Trauma Surgery, University Hospital Regensburg, Regensburg, Germany; ^2^Institute of Clinical Microbiology and Hygiene, University Hospital Regensburg, Regensburg, Germany; ^3^Laboratory for Molecular and Cellular Technology (LabMCT), Queen Astrid Military Hospital, Brussels, Belgium; ^4^Department of Infection Prevention and Infectious Diseases, University Hospital Regensburg, Regensburg, Germany

**Keywords:** bacteriophage, fracture-related infection, metagenomic, Masquelet, hydrogel

## Abstract

Fracture-related infections can be challenging, particularly with concomitant severe bone defects and multi-resistant microorganisms. We present a case of a 42-year-old patient with a fracture-related infection following a war injury from a gunshot, resulting in a 12-cm subtrochanteric segmental bone defect and the detection of four different multi-resistant Gram-negative bacteria. Due to antibiotic drug resistance, treatment with bacteriophages was considered. Phage susceptibility testing revealed the activity of a commercially available bacteriophage cocktail (Intesti bacteriophage, Eliava Institute, Tbilisi, Georgia). This phage cocktail was included in a modified two-stage Masquelet technique. During the first intervention, the bone was debrided and samples for microbiological and phage testing were harvested. The indwelling intramedullary rod was removed, and the bone defect was filled with a PMMA spacer loaded with colistin and the bone stabilized with a plate. During the second procedure, the PMMA spacer was removed and a silver-coated angular stable plate was implanted. The bone defect was filled with a fibular autograft and allograft cancellous bone chips. At the end of the procedure, the Intesti bacteriophage cocktail was injected into a DAC hydrogel and this bacteriophage hydrogel composite was then put onto the angular stable plate. Postoperatively the wound fluid was collected over 72 h, and high-throughput metagenomic sequencing was performed. This showed a time-dependent release of the bacteriophages in the wound fluid, with a relatively high concentration after 12 h, decreasing to DNA copies of 0 after 72 h. Furthermore, we have assessed the release of phages from DAC gel and the effect of DAC gel on the phages *in vitro*. The results showed a stable and rapid release of phages from the DAC gel (~1×10^3^ PFU/mL). The clinical course of the patient showed no relapse of the infection with good bone consolidation of the bone defect after 1 year without the need for any surgical revision. To the best of our knowledge, this is the first case that shows the detection of bacteriophage DNA copies by high-throughput metagenomics sequencing in a patient with a complex fracture-related infection. Successful treatment of this case encourages further investigation of bacteriophage therapy in patients with complex bone and joint infections.

## 1 Introduction

The treatment of fracture-related infections (FRI) is challenging. Particularly, the involvement of severe bone defects and multi-resistant bacteria frequently leads to complex situations. The treatment of FRIs mainly consists of two key aspects: First, control of the infection and causative microorganisms must be achieved. Second, bone reconstruction with subsequent successful bone healing needs to be obtained (1).

Both local and systemic antibiotic therapy are hallmarks of fracture-related infection treatment. However, there are often only limited antibiotic treatment options when multi-drug resistant bacteria are involved. An alternative or complementary approach for the treatment of complex infections with multi-drug resistant bacteria is bacteriophages (short phages). The bactericidal effect of phages was first identified more than 100 years ago by Felix d’Herelle (2). Since then, phages have been studied and used mainly in the former Soviet Union during the second half of the 20th century. In recent years, bacteriophage therapy has also gained interest in the Western world due to the rise of multi- and pan-drug-resistant bacteria, including difficult-to-treat bone and joint infections (3). However, there are only a few published case series or case reports on the treatment of musculoskeletal infections with bacteriophages (4). A recent review identified 33 bone and joint infection cases treated with phages, out of which notably 29 (87%) achieved either microbiological or clinical success. Furthermore, 8 of the 33 cases (24%) experienced mild and temporary adverse effects, but there were no reports of serious complications (5). In addition, underlining the efficacy of phage therapy in resolving difficult-to-treat infections, Pirnay et al. have just recently published clinical outcomes of their first 100 cases treated with personalized phage therapy. Bone infections were the three most common indications. Overall, in 77.2% of targeted infections, patients experienced clinical improvement. In 61.3% of infections where relevant bacteriological follow-up data were available, eradication of the targeted bacteria was observed (6). Ongoing clinical trials aim to refine phage application mode and advance our understanding of how to effectively manage bone infections. Currently there are a few clinical trials registered, which already entered the phase of recruiting patients for phage treatment.

Hitherto, however, the optimal administration of bacteriophages has not yet been elucidated. Phages can be administered intravenously or locally. Different approaches with varying quantity, frequency, and type of application have been described to date (4, 7). Recently, Ferry et al. published a case report with the use of hydrogel as carrier material for the application of bacteriophages in a patient with a mega prosthetic hip infection (8). This study showed a rapid release of the phages from the DAC hydrogel in an *in vitro* setting. However, no *in vivo* data for the release of the phages in the patient were presented.

Therefore, in this study, we present a clinical case involving the loading of bacteriophages into a hydrogel in an FRI. Additionally, we assess the *in vivo* release kinetics of the phages from the hydrogel in the patient’s wound using high-throughput metagenomic sequencing-based characterization of the administered bacteriophage cocktail, followed by real-time PCR quantification of selected bacteriophages in the drainage fluid.

## 2 Case description

**Figure 1:**
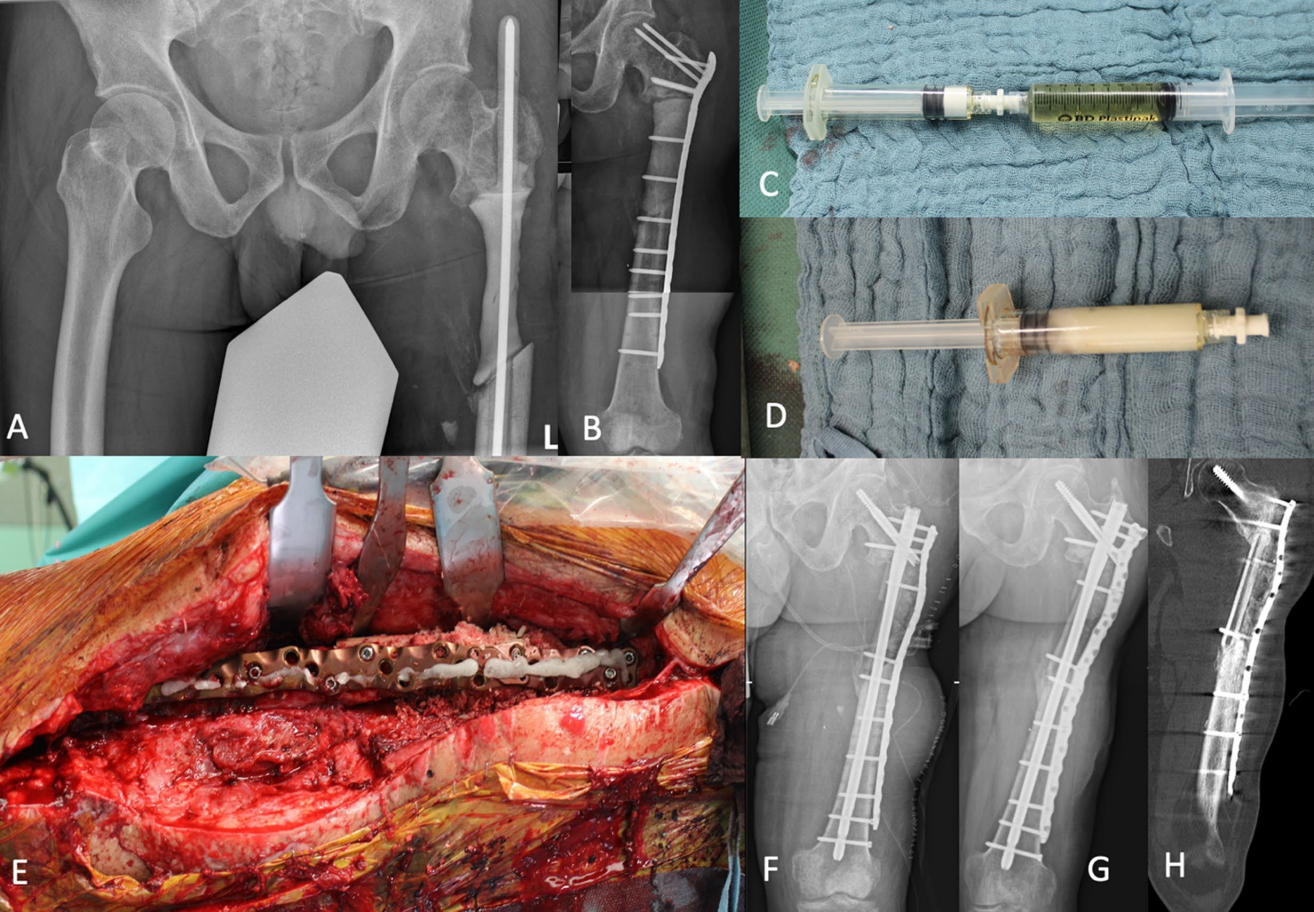
Subtrochanteric segmental bone defect of the left femur was temporarily stabilized with an intramedullary custom-made PMMA bone cement-coated rod **(A)**. After removal of the rod, a PMMA spacer with colistin was added for dead space management and the bone stabilized with an angular stable plate **(B)**. The second stage of the Masquelet procedure was performed with an intramedullary nailing and augmentation plate. During bone defect reconstruction phages were applied to the DAC® gel **(C,D)**. The DAC gel loaded with Intesti bacteriophage was finally placed on the silver-coated plate **(E)**. Postoperative X-ray demonstrates the bone defect filled with autograft fibula graft and allogenic cancellous bone **(F)**. After 12 months, sufficient bone healing was confirmed by performing X-ray **(G)** and computed tomography **(H)**.

A 42-year-old man suffered a war injury during the Russian war in Ukraine. He had a history of a gunshot and a blast injury to his proximal left femur 8 months ago. He further sustained an open head injury and a fracture of the 3rd metacarpal of his left hand. The injury of the proximal femur also included a lesion of the sciatic nerve, mainly affecting the peroneal part. The patient has been treated in Ukraine with several debridement and irrigation procedures of the left femur, along with the application of a ring fixator. Multiple revision surgeries for debridement, and irrigation of local antibiotics resulted in a 12-cm-wide segmental subtrochanteric bone defect. After the transferal to Germany, the external fixator was removed, and the implantation of an antibiotic-coated intramedullary rod was performed. The microbiological results of this revision surgery revealed several multi-drug resistant pathogens such as multi-drug resistant (MDR) *Pseudomonas aeruginosa*, MDR *Proteus mirabilis*, MDR *Klebsiella pneumoniae*, MDR *Escherichia coli,* and vancomycin-resistant *Enterococcus faecium*. A combination therapy with daptomycin, cefiderocol, and fosfomycin was established. After presentation in our department, a modified two-staged Masquelet procedure was performed with the removal of the antibiotic-coated rod and debridement of the subtrochanteric bone defect. Microbiological analysis of the tissue samples harvested during the first Masquelet procedure surgery revealed *Cutibacterium acnes,* which was sensitive to penicillin G, vancomycin, and clindamycin. In addition, *S. epidermidis* susceptible to vancomycin, rifampin, and cotrimoxazole was detected. The previously cultivated multi-resistant Gram-negative bacteria could not be detected. The 12-cm segmental bone defect was managed with a daptomycin- and colistin-loaded polymethyl methacrylate (PMMA) spacer (80 g Copal® (Heraeus Medical, Germany) + 6 Mio. I.E colistin/CMS and 2 g daptomycin), and proximal femur was stabilized with an angular stable plate (NCB, Zimmer Biomet, USA). Systemic antibiotic therapy was initially performed with fosfomycin 3x5g, colistin (CMS) 3x3MioIU, and daptomycin 1x1g and administered until final surgery. Cultures of the initially evidenced MDR pathogens were sent to the Queen Astrid Military Hospital, Brussels, Belgium, for phage susceptibility testing. After testing the available phages, the best possible option for additional treatment seemed Intesti bacteriophage cocktail (Eliava Institute, Tbilisi, Georgia). The phage cocktail then was used with a hydrogel carrier to improve the release kinetics of this composite biomaterial, as described in a recently published case by Ferry et al. (8). After 92 days during which phages were tested and wound healing was achieved, the final surgery for bone defect reconstruction was performed. The patient was informed about the intended compassionate use of phages according to §37 of the Helsinki Declaration, and written consent was obtained (9). The indwelling PMMA spacer and the angular stable plate were removed, and the bone ends were debrided again. Five tissue samples were sent for microbiological analysis, which revealed no detectable pathogen. Perioperative antibiotic therapy with daptomycin and meropenem i.v. was supplemented with colistin i.v. for 14 weeks postoperatively and finished after negative long-term incubation. There was no sign of persistent infection, and the decision was taken to perform a bone reconstruction with an ipsilateral autologous fibula graft and allogenous cancellous bone within a nicely formed Masquelet membrane. The subtrochanteric bone region was stabilized by a combination of a long proximal femur nail (Gamma nail, Stryker, USA) together with a silver-coated (HyProtect™ coating, Bio-Gate, Germany) angular stable plate (NCB Zimmer Biomet, USA). The angular stable plate was slightly bent in order to allow a high volume of bone grafting of the defect with a subsequent spindle-shaped callus formation. The Defensive Antibacterial Coating (DAC®, Novagenit, Italy) gel loaded with bacteriophage cocktail was applied onto the plate before wound closure (Figure 1).

Wound drainage fluid and serum samples taken after 12, 24, 48, and 72 h were evaluated regarding the release of bacteriophages from the hydrogel into the wound.

The patient was then followed up for 12 months. The wound healed uneventfully, and there was no sign of reinfection, nor was there a need for further surgical revision. X-rays and computed tomography demonstrated sufficient bone healing of the grafted area, achieving bone consolidation after 12 months.

## 3 Materials and methods

### 3.1 Testing activity of phages on the patient strains

The activity of phages was tested by spot-test using 100-fold dilutions of Intesti bacteriophage cocktail (The Eliava IBMV), individual phages, phages produced as active pharmaceutical ingredients (API) available from the collection of Queen Astrid Military Hospital, Brussels, Belgium. Intesti bacteriophage is a mix of sterile filtrate of phage lysates active against *Shigella* spp. (*Shigella flexneri* serotype 1,2, 3, 4 and *Shigella sonnei*) (titer no less than 10^5^ mL^−1^), *Salmonella* spp. (*S. patarype A, S. paratype B, S. typhimurium, S. enteritidis, S. cholera suis, S. oranienburg*) (titer no less than 10^5^ mL^−1^), different types of *E. coli* (titer no less than 10^5^ mL^−1^), *Proteus mirabilis* and *Proteus vulgaris* (titer no less than 10^5^ mL-^1^), *Staphylococcus aureus* (titer no less than 10^5^ mL^−1^), *Pseudomonas aeruginosa* (titer no less than 10^5^ mL^−1^), and *Enterococcus* spp. (titer no less than 10^5^ mL^−1^). Inactive ingredients are bacterial growth media, standard sodium saline, and Chinazolin as a preservative. Phage APIs have quality control certificates from Sciensano (formerly known as the Belgian Scientific Institute of Public Health) and are ready-to-use pharmaceutical ingredients to be used in magistral phage preparations (10). Shortly, each phage was diluted 100 times in DPBS and 10 μL of each dilution was spotted on the lawn of bacterial strains made with semi-solid 0.6% lysogenic broth (LB) agar as a second layer on the surface of LB agar in square Petri plates. The starting concentration of each phage except Intesti bacteriophage was 10^9^ plaque-forming units per milliliter (PFU/mL). The results were recorded after overnight incubation at 32°C. Individual plaques were counted where visible and efficiency of plating (EOP) was defined as a ratio of the titers of phage on the test strain and the host strain.

### 3.2 High-throughput metagenomic sequencing

In order to analyze the composition of the Intesti bacteriophage cocktail, metagenomic DNA was extracted from 2 mL of the original suspension before administration. DNA was extracted following the protocol of Shkoporov et al. (11). Briefly, viral particles were precipitated by treatment with polyethylene glycol followed by DNAse and RNAse digestion of free nucleic acids. DNA was extracted from the precipitate containing bacteriophages using phenol/chloroform extraction. Sequencing was performed on the original bacteriophage suspension. For this purpose, DNA was fragmented to ~150 bp using the M-220 focused ultrasonicator (Covaris, Woburn MA, USA) followed by two-sided size selection with MagSi-NGSPREP-PLUS beads (magtivio, Nuth, The Netherlands) using a DNA to bead ratio of 1:1.1 and 1:0.6. The Ion Plus Fragment Library Kit (Thermo Fisher Scientific, Whatman, MA, USA) was used to prepare the sequencing libraries. The final library was quantified using the Ion Library TaqMan™ Quantitation Kit (Thermo Fisher Scientific, Whatman, MA, USA) and diluted to a concentration of 70 pM. The sequencing library was amplified using the Ion 550™ Kit on an Ion Chef instrument and loaded onto a 550 chip. High-throughput sequencing was performed on an Ion GeneStudio™ S5 Plus instrument, resulting in a total of 3.9 million reads with an average read length of 161 bp.

### 3.3 Analysis of viral metagenomic sequencing data

Raw sequencing reads were obtained from the Torrent Suite v5.18.1 instrument software and then quality-filtered using Trimmomatic v0.9 (12). Sequencing adapters were removed using cutadapt v4.0 (13). Reads mapping to the human GRCh37 reference genome were removed using bowtie2 v.2.5.0 and samtools v1.17 (14, 15). Filtered reads were assembled using megahit v1.2.9. The VIRify pipeline was used to detect and classify viral contigs (16). Sourmash v4.8.0 was used for viral taxonomic classification of viral contigs by MinHash sketching and searching against the viral genbank 2022.03 database (17). Here, a k-mer length of 31 and a scaled value of 1,000 were used for calculating signatures from assembled viral contigs. Default parameters were used for taxonomic classification using the Sourmash tax algorithm.

### 3.4 Quantification of bacteriophage DNA in drainage fluid

In order to quantify viral bacteriophage genomes in wound drainage fluid or blood, a total of 10 mL sample material was taken at five different time points after surgery: immediately after surgery, 12 h post-surgery, and on days 1, 2, and 3 after surgery. DNA extraction was executed as described above. The two largest contigs, which matched to *Proteus* and *Pseudomonas* bacteriophage genomes, were selected to quantify bacteriophage DNA copies from the original bacteriophage suspension and drainage fluids collected at different times: First 12 h, 12 h, and 1, 2, and 3 days post-operatively. Primers S12_k141_521_f (5’-TATCTATCCCTCTCCCGCCG-3′) and S12_k141_521_r (5’-GTTGAAGATAACGCCGACGC-3′) were used to detect the viral contig VC_521 (Pseudomonas phage KPP10). Primers S12_k141_755_f (5’-AGTGTGTACAGAGCCAGTGC-3′) and S12_k141_755_r (5’-GCGGTATCACCAGCTAGCAT-5′) were designed to target a fragment of viral contig VC_755 (Proteus phage vB_PmiP_RS1pmA). Fragments cloned into the pJET 1.2 cloning vector (Thermo Fisher Scientific, Whatman, MA, USA) were used to quantify bacteriophage DNA from nucleic acid extracts on a LightCycler 480 II using the LightCycler® 480 SYBR Green I Master (Roche Molecular Sciences, Rotkreuz, CH).

### 3.5 The release of pages and DAC gel effect on the phages *in vitro*

To analyze the release of the phages from DAC gel and the effect of the DAC gel on the phages, we have incubated the DAC gel loaded with Intesti bacteriophage in PBS at 37°C. The activity of the phages was tested against the *E. coli* at different time points (0 h, 0.5 h, 1 h, 2 h, 4 h, and 6 h) using double-layer agar method.

## 4 Results

All five strains of different species were tested against the phage collection of Queen Astrid Military Hospital, Brussels, Belgium, and the Intesti bacteriophage cocktail produced by the Eliava IBMV. In particular, *P. aeruginosa* strain was tested against 6 phage APIs and 18 individual phages. Only four individual phages proved to be active with EOP in the range of 1.0–0.01; three active phages were representatives of the *Pakpunavirus* species. The nomenclature affiliation of the fourth phage was not known.

*E. faecium* strain was tested against 1 API and 4 individual phages. *K. pneumoniae* strain was tested against 27 individual phages. *E. coli* tested was tested against 5 individual phages. None of the APIs or individual phages showed any activity on the above three strains. *P. mirabilis* was tested only with an Intesti bacteriophage cocktail. Intesti bacteriophage showed activity only against the *E. coli* strain. The titer of the phage(s) in the cocktail active against the *E. coli* strain of the patient was defined as 2 ×10^3^ PFU/mL.

As no active phage API was available against the bacterial strains and the patient needed to be treated urgently, it was decided to start treatment with Intesti bacteriophage, as the only available option at that time.

### 4.1 *In vivo* assessment of bacteriophage release from hydrogel

**Figure 2:**
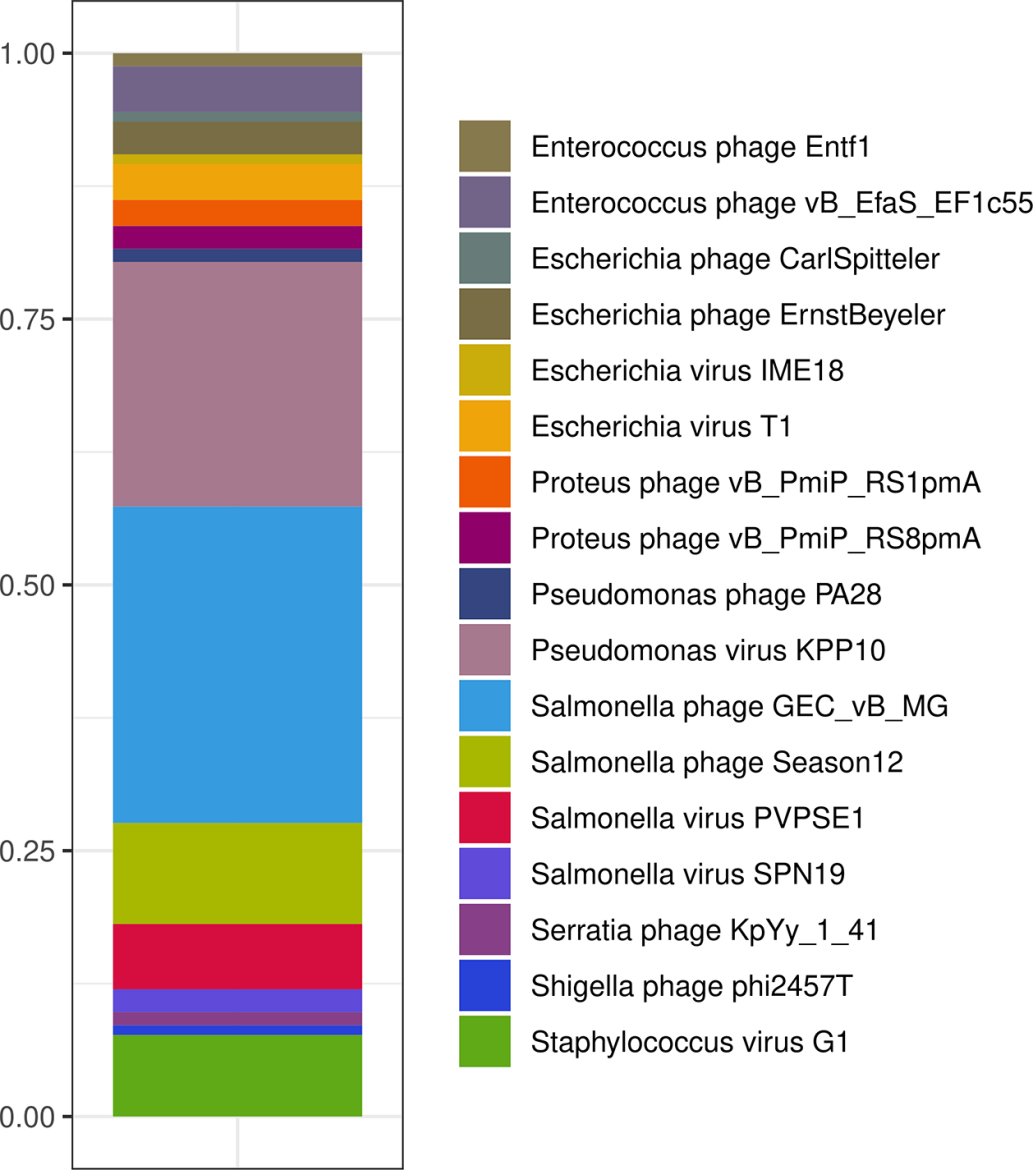
Composition of the Intesti bacteriophage cocktail as revealed by whole shotgun metagenomic sequencing. Viral contigs were analyzed by k-mer-based searching against the GenBank nt database.

In order to characterize the composition of bacteriophage genomes within the Intesti bacteriophage suspension, nucleic acids were extracted directly from the administered bacteriophage suspension using whole shotgun metagenomic sequencing. A total of 511,078 (72,7 Mbp) raw sequencing reads were assembled to 149 contigs (N50 = 9,914 bp). Of these, 77 could be identified as viral contigs using the VIRify pipeline. The taxonomic assignment resulted in 10 different bacteriophage genera, which comprised *Seunavirus, Saphexavirus, Nankokuvirus, Kayvirus, Tunavirus, Hanrivervirus, Chivirus, Bruynoghevirus, Teseptimavirus,* and unclassified Casjensviridae. Further k-mer-based searching against the GenBank nt database-matched bacteriophages that target bacterial hosts *Staphylococcus, Enterococcus, Proteus, Shigella, Salmonella, Escherichia coli, and Pseudomonas* (Figure 2). This was fully in line with the list of bacterial hosts in the Intesti bacteriophage package insert.

**Figure 3:**
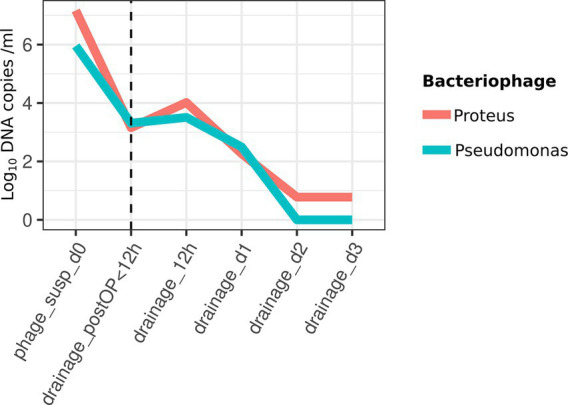
Quantification of bacteriophage release by real-time PCR targeting viral contigs encoding a Proteus and Pseudomonas targeting bacteriophage in postoperative drainage fluid as well as in Intesti bacteriophage suspension (viral copies per mL). Samples were collected at 5 different time points: immediately after surgery, 12 h post-surgery, and on days 1, 2, and 3. The time point of surgery is marked with a dashed line.

To measure the release of bacteriophages from hydrogel in drainage samples, specific real-time quantitative PCR protocols were developed. These protocols were based on two selected metagenomic viral contigs, identified through k-mer-based searching against the GenBank nt database, which presumably encodes bacteriophages targeting bacterial species of the genus *Proteus* and *Pseudomonas*. Genomic copies of bacteriophage from the Intesti bacteriophage cocktail prior to administration and from drainage samples taken at 5 different time points after surgery were quantified by qPCR: immediately after surgery, 12 h post-surgery, and on days 1, 2, and 3 after surgery. The analyses revealed that bacteriophages were released from the hydrogel within the first 3 days following surgery (Figure 3). No DNA of these two bacteriophages was detected by qPCR in the blood samples taken at the same time points.

### 4.2 *In vitro* assessment of bacteriophage release from DAC hydrogel

**Figure 4:**
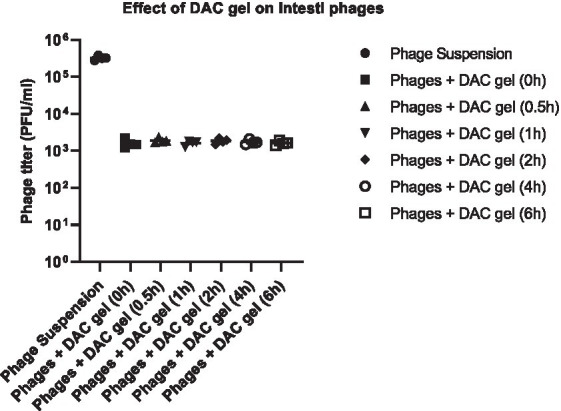
The release of bacteriophages from hydrogel and the impact of DAC gel on phages. The scatter dot plot graph was created using GraphPad Prism 9.5.

The release kinetics of the DAC hydrogel injected with Intesti bacteriophages showed the rapid release of the phages from hydrogel after incubation in PBS. There were no differences in the release of bacteriophages with the time progress (0 h-6 h) at 37°C incubation. The released phage titer was between 1.6 × 10^3^ and 1.9 × 10^3^ PFU/mL, which indicates a fairly consistent release rate of the phages from the DAC gel (Figure 4).

## 5 Discussion

In the present case of a Ukrainian soldier wounded in the Russian-Ukrainian war, a combination of local bacteriophage therapy with established therapeutic procedures achieved infection eradication and bone reconstruction of the injured leg despite chronic osteomyelitis caused by multiple MDR bacteria. *In vitro* analysis evidenced a consistent release of phage from the DAC hydrogel. Furthermore, phage analysis conducted in the postoperative course demonstrated the activity of phages in the wound area during the first postoperative days, with no systemic spread of phages through the blood detectable within the initial 3 postoperative days. No side effects of phage therapy were observed.

In orthopedic trauma surgical treatment, the local application of phages appears promising. However, the optimal form of application, required quantities, and the frequency of phage application remain unclear (4). While some authors advise the use of a draining device for the administration of phages into the wound cavities, the potential of the hydrogel as a phage delivery system has been highlighted (6, 18). *In vitro* and *in vivo* results of the present case indicate that application via a hydrogel may be a possible and practical solution. These findings are in line with a previously published case reporting a salvage therapy in a patient with an infected knee megaprosthesis. The *in vitro* testing of the impact of the hydrogel on phage activity showed a rapid release and also stable titers for at least 6 h indicating compatibility (8). The present case shows that this innovative approach is also feasible for the treatment of bone infection and that phages delivered via hydrogel may offer a promising strategy by targeting specific bacterial strains. Based on these results, localized and sustained delivery of phages to the infection site could be demonstrated. Furthermore, it can be assumed that the efficacy of phage therapy may be enhanced by maximizing contact time between phages and bacteria compared to rinsing the wound cavities with phages. Another potential implication involves the versatility of hydrogel delivery systems, which enable the incorporation of other therapeutic agents, such as antibiotics for a synergistic approach.

Metagenomic sequencing is a powerful tool for identifying and characterizing bacteriophages. However, its analytical sensitivity poses challenges in detecting low-abundance bacteriophages directly from the original clinical sample material (19). Diluting effects are particularly important for high-volume sample material such as wound drainage fluid or blood. Additionally, the complexity of these samples, combined with the presence of host DNA, can mask bacteriophages. Therefore, we used specific real-time quantitative PCR (qPCR) protocols to confirm the presence of phages, ensuring sensitive detection of bacteriophages. Reduction in bacterial counts and the stability of phage titers as well as qPCR protocols are commonly employed to confirm the presence and activity of phages post-release and from hydrogels (20). These findings are in line with other studies that detected the release of bacteriophages from hydrogels for several days (21). Nevertheless, detection will depend on the sensitivity of the detection method and the sample material. Furthermore, the development and standardization of phage cocktails pose several challenges. This study highlights the need for rigorous characterization to ensure the safety and efficacy of these cocktails.

The case presentation also highlights practical weaknesses in the current therapeutic approach and reveals challenges to be addressed. (1) In three performed revision surgeries, different communities of antibiotic-resistant microbes were observed in routine diagnostics conducted through culture in each operation. This can often be observed in bone and joint infections (22, 23). However, the therapeutic consequences for antibiotic and alternative therapies, such as phage application, still need to be determined. Very high local antibiotic doses for MDR bacteria may lead to the initial disappearance of MDR bacteria and eventual infection eradication (24). (2) Bacteriophage testing on the identified MDR bacteria did not yield the initially desired results, except for the Georgian phage cocktail, which demonstrated efficacy against MDR *E. coli*. (3) The time elapsed until obtaining results was substantial. While a 6-week interval between operations is typically required for a two-stage Masquelet procedure, in this case, the therapeutic interval of 92 days is more than twice as long. Therefore, the testing and production of suitable phages must be significantly optimized.

## 6 Conclusion

In conclusion, this case demonstrates the potential of localized phage therapy delivered via hydrogel in managing complex fracture-related infections with multi-drug resistant bacteria and severe bone defects. The integration of phage therapy with established surgical procedures resulted in successful infection eradication and bone reconstruction. High-throughput metagenomic sequencing provided insights into bacteriophage release dynamics in the wound environment, supporting the feasibility of sustained delivery for several days after surgery in a clinical context. The case study highlights the fact that applying phages had no negative side effects. It remains uncertain in the present case whether bacteriophage application has effectively contributed to preventing reinfection. Despite practical challenges, including microbial variability and timely production processes, phage therapy holds promise as a personalized approach for patients with challenging bone infections.

## Data availability statement

The raw data supporting the conclusions of this article will be made available by the authors, without undue reservation.

## Ethics statement

Ethical approval was not required for the studies involving humans because this case falls under the category of intended compassionate use of phages according to the §37 of the Helsinki Declaration. The studies were conducted in accordance with the local legislation and institutional requirements. The participants provided their written informed consent to participate in this study. Written informed consent was obtained from the participant/patient(s) for the publication of this case report.

## Author contributions

VA: Conceptualization, Data curation, Formal analysis, Investigation, Methodology, Validation, Writing – original draft. AG: Conceptualization, Data curation, Formal analysis, Investigation, Methodology, Project administration, Validation, Writing – review & editing. MM: Formal analysis, Investigation, Methodology, Validation, Writing – review & editing. FH: Investigation, Methodology, Validation, Writing – review & editing. GM: Data curation, Formal analysis, Investigation, Methodology, Validation, Writing – review & editing. DP: Formal analysis, Investigation, Methodology, Validation, Writing – review & editing. NW: Conceptualization, Investigation, Validation, Writing – review & editing. J-PP: Investigation, Methodology, Validation, Writing – review & editing. AH: Formal analysis, Investigation, Methodology, Validation, Writing – review & editing. MR: Conceptualization, Data curation, Investigation, Methodology, Project administration, Validation, Writing – original draft.
